# Assessing the factor structure of the Spanish language parent Strengths and Difficulties Questionnaire (SDQ) in Honduras

**DOI:** 10.1371/journal.pone.0214394

**Published:** 2019-03-28

**Authors:** Melissa L. Harry, José Acevedo, Thomas M. Crea

**Affiliations:** 1 Essentia Institute of Rural Health, Essentia Health, Duluth, Minnesota, United States of America; 2 School of Sociology, National Autonomous University of Honduras, Tegucigalpa, Honduras; 3 School of Social Work, Boston College, Chestnut Hill, Massachusetts, United States of America; University of La Rioja, SPAIN

## Abstract

With worldwide prevalence rates between 10% and 20%, mental illness in children and adolescents is an issue for which culturally sensitive screening tools are needed. The Strengths & Difficulties Questionnaire (SDQ) is a commonly used measure that has been translated into numerous languages, although some research suggests issues with cross-cultural validity. Only four other studies have tested the Spanish-language SDQ in Latin America. In this study, we aimed to help fill this gap by assessing the factor structure of the parent or teacher version of the Spanish-language SDQ (for children ages 4–17) with 967 parent or other caregiver respondents of primary school-aged children (ages 4 to 17) in the Department of Intibucá, Honduras. When unable to find a good fitting factor model previously identified in the literature, we conducted split sample exploratory factor analysis (EFA) and confirmatory factor analysis (CFA), along with measurement invariance testing with the best fitting EFA-extracted model based on gender for caregiver respondent and child gender. Results showed that while many EFA models had a good fit, CFI and TLI was < .90 for all extracted models when confirmed in the second sample with CFA. We then modified the best fitting extracted three-factor, 24-item model, which dropped item 15, by allowing select item residuals to correlate, increasing CFI and TLI to > .90 for female child gender. This modified three-factor model was partially invariant for configural and scalar models between child genders. Configural and scalar models would not converge for adult genders. Of note, metric models were not produced in Mplus related to items’ cross-loading on more than one factor. These findings suggest that the SDQ Spanish language parent or teacher version may not perform optimally cross-culturally in this area of Honduras. Future research should confirm these findings with other Honduran populations.

## Introduction

Child and adolescent mental and behavioral health are a global concern. Research has shown that the prevalence of mental health disorders in children and adolescents ranges from 10 to 20% worldwide [1, 2], and may affect approximately 1 in 5 people over their lifetimes [3]. Furthermore, mental illness may account for more disability burden than previously thought as recent estimates suggest mental illness alone accounts for 32.4% of years lived with disability [4]. More needs to be done to identify and treat children and adolescents at risk of mental illness, particularly in low- and middle-income countries where such resources are scarce [1, 5]. Given the potential for cultural variations in symptomology and stigma related to experiencing mental illness, culturally relevant and evidence-based treatments [6], as well as screening tools, are needed. Culturally sensitive screening for both protective and risk-related factors, which can co-occur together to varying degrees over the lifespan [1], is an important first step for identifying children needing mental health care.

### The Strengths and Difficulties Questionnaire

The Strengths and Difficulties Questionnaire (SDQ) [7] is a widely used tool for assessing and screening children’s psychosocial functioning that has been translated into numerous languages and regional dialects (see www.sdqinfo.com). The SDQ measures five subscales: Emotional Symptoms, Hyperactivity, Peer Problems, Conduct Problems, and Prosocial Behavior. The SDQ was developed in the United Kingdom and initially tested in a sample of children from London [7]. Further studies support the use of the SDQ with children in the UK. A national epidemiological sample of children ages 5–15 revealed strong support for the five-factor structure and little overlap between items on the internalizing and externalizing scales, thereby verifying that the two are uncontaminated by one another [8]. A subsequent study that looked at the dimensionality of the SDQ and the accuracy of correlations between SDQ scores and rates of mental illness in children aged 5–16 found that higher difficulty scores are associated with greater psychopathology for parent, teacher, and child versions of the SDQ [9]. Croft and colleagues supported the use of the five-factor SDQ for identifying emotional and behavioral disturbances in preschool-aged children in the UK [10]. Another study of adolescents in Australia found the SDQ to have convergent and discriminant validity between adolescents, parents, and teachers [11]. Goodman and colleagues also found that the standard five-factor SDQ had a better fit for British parents, teachers, and children ages 6–18 than two theoretically-supported models (a five-factor model with second-order Externalizing and Internalizing factors and a three-factor model where Hyperactivity subscale items were included with Conduct Problems items in a Behavioral factor and Emotional Symptoms and Peer Problems items with an Internalizing factor) [12]. However, they reported that the five-factor, second-order model was feasible to use in samples of children at low risk of mental health issues, while the standard five-factor SDQ was more appropriate for diagnostic use [12].

### The cross-cultural validity of the SDQ

Some studies of children in the United States have also found that the SDQ is a valid screening measure for children’s mental health. One study of children aged 4–17 found similar scores to those of British children and good internal consistency [13]. Another study using a nationally representative sample of U.S. adolescents aged 13–18 revealed a satisfactory fit for the five-factor structure that remained stable across subgroups [14]. However, in a sample of first grade children, Hill and Hughes found a marginal fit for the five-factor structure of the SDQ [15]. Furthermore, Dickey and Blumberg found that a three-factor SDQ model with Internalizing, Externalizing, and Prosocial Behavior factors, like the first-order three-factor model later tested by Goodman et al. [12], was the best fit with the parents or guardians of American children and adolescents [16]. When testing the SDQ with custodial grandparents, Palmieri and Smith found that a model that included a positive construal method factor encompassing reverse-coded items and the Prosocial Factor had the best fit [17].

The SDQ appears to be an effective screening instrument in other Western European countries. In Germany, Becker et al. replicated the five-factor structure of the SDQ and demonstrated a high degree of correlation with the Child Behavior Checklist [18]. Similarly, DeVries et al. demonstrated good model fit and measurement invariance across age groups and over time, although the authors assert that more research is needed in multicultural settings where inconsistent measurement invariance has not been accounted for [19]. Petermann et al. also found the SDQ to exhibit good validity for the assessment of behavior in preschool-aged children in Germany [20]. Similar results have been demonstrated for the Danish [21, 22], Norwegian [23–25], and Swedish [26] versions of the SDQ. The Dutch version has also demonstrated strong validity [27, 28]. Furthermore, in a sample of Dutch parents, Stone et al. reported SDQ subscales had adequate to very good internal consistency (0.74 for Conduct Problems, 0.79 for Peer Problems, 0.82 for Emotional Symptoms, 0.85 for Prosocial Behavior, and 0.91 for Hyperactivity) using McDonald’s omega (ω) [29, 30]. Stone et al. advise using McDonald’s ω rather than Cronbach’s alpha when evaluating the SDQ [29]. However, one study showed marginal internal consistency for the SDQ subscales (except for the Total Difficulties score) and found the measure to be more sensitive to externalizing problems than internalizing problems in preschool-aged children [31]. Another study of Dutch adolescents aged 11 to 16 showed that allowed reverse-coded SDQ items to also load on the Prosocial factor gave a better fit than the standard five-factor model, as did a four-factor model that combined Emotional Symptoms and Peer Problems factors into a single factor [32].

Two studies were conducted with data from the Spanish National Health Survey to determine the efficacy of the Spanish version of SDQ, one with children from the 2006 survey [33], and the other with parents from the 2011–2012 survey [34]. Results from the first study with children between the ages of 4–15 indicated adequate diagnostic efficiency and acceptable goodness of fit for both three- and five-factor models [33]. The second study found the five-factor model to be an acceptable fit when correlating residuals for the parent version of the SDQ in Spain, but not the three-factor model [34]. When assessing the adolescent self-report SDQ in adolescents in Spain, Ortuño-Sierra and colleagues reported that while both the five-factor model and a bifactor model with correlating errors showed acceptable fits, the five-factor model had the best fit overall [35]. However, a study conducted with the Hungarian parent or teacher version of the SDQ found that a bifactor model was the best fit for parents and teachers, including compared to the five-factor model [36]. Bifactor models, originally reported by Holzinger and Swineford [37], assume an overarching general latent factor, or dimension, upon which all items in a scale load in addition to loading on subfactors. Also, Di Riso et al. found good internal consistency for the Total Difficulties score and poor reliability for the self-report SDQ subscales for Italian children ages 8–10, a finding which the authors attributed to different child-rearing and educational practices compared to other Western European countries [38].

As noted above, several studies have examined the factor structure of the SDQ and established its validity and reliability, but some evidence suggests that translated versions of the SDQ do not function as expected in some cultural contexts. Specifically, some evidence suggests that its five-factor structure may not function as expected cross-culturally. While the SDQ did have good overall ordinal alpha internal consistency with samples from five European countries (England, France, Germany, Ireland, and Spain), some subscales showed inadequate levels of internal consistency, specifically the Peer Problems subscale in Ireland (0.61) and Conduct Problems subscale in France and Spain (0.61, 0.68, respectively) [39]. Furthermore, Ortuño-Sierra and colleagues conducted measurement invariance testing and found that the standard five-factor SDQ solution was only partially invariant across samples from these five countries, with variance seen in 11 of the 25 SDQ items [39]. Other tested models that included correlating residuals and cross-loading reverse-coded items on the Prosocial Behavior factor did provide adequate fits in measurement invariance testing [39], a model similar to that tested by Palmieri and Smith [17]. A systematic review of 41 studies examining the psychometric properties of the SDQ demonstrated evidence in support of the five-factor model and good convergent validity [40]. Internal consistency was strong for the total Difficulties scale, but weaker for the other subscales, and some issues were identified in terms of cultural validity [40]. Stevanovic and colleagues performed exploratory structural equation modeling (ESEM) when unable to find a good-fitting SDQ model for measurement invariance testing among different countries (India, Indonesia, Nigeria, Serbia, Turkey, Bulgaria, and Croatia), finding that only the Prosocial Behavior, Emotional Symptoms, and Conduct Problems subscales were reproduced between countries studied [41]. Goodman et al. did note that “there may be no single best set of subscales to use in the SDQ; rather, the optimal choice may depend in part upon one’s study population and study aims” (p. 1189) [12].

Studies of the cross-cultural application of the SDQ in Asian cultures have revealed mixed results. In a study of children ages 3–17 in twelve administrative districts in China, Du, Kou, and Coghill found low internal consistency with parent and teacher Hyperactivity and Prosocial Behavior subscales and lower than expected test-retest reliability, which the authors attributed to different cultural interpretations of the questions, and greater cross-cultural acceptance and consistency of behaviors characterized as prosocial or hyperactive/impulsive [42]. Another study in China with children ages 5–13 found satisfactory results in four of the five subscales, but low internal consistency within the Peer Problems subscale, and discrepancies between children from urban and rural areas [43]. Kersten et al. did note that the Chinese version of the SDQ appeared to require further translation [40]. A study conducted with parents of Singaporean kindergarteners investigated the fit of three proposed models for the SDQ and found the best fitting model to include four trait factors and two method factors [44]. While the researchers promoted the use of the SDQ in Singaporean communities, they also advised caution when comparing scores across gender and countries [44]. A study in Japan of children aged 4–12 produced favorable psychometric properties comparable to the original English version of the SDQ [45]. The results indicated that boys scored higher than girls on the Total Difficulties score [45]. Also, Gomez and Stavropoulos found that a six-factor model with a positive construal factor encompassing the reverse-coded items and Prosocial Behavior factor was the best fit for Malaysian parents [46]. They also noted that the standard five-factor model showed a good fit, and that all 12 models tested had adequate fits with the sample of Malaysian parents [46]. However, Stokes and colleagues failed to validate previously identified SDQ factor structures with a sample of Malaysian children, parent, and teacher triads; rather, the authors conducted a split sample exploratory factor analysis (EFA), producing a three-factor structure, which was partially supported in confirmatory factor analysis (CFA) [47].

Two studies that produced less favorable results were those conducted using the Urdu and Arabic versions of the SDQ. The Urdu version of the SDQ showed good discriminant validity and sensitivity with children and adolescents aged 4–16, but results illustrated inaccurate screening of control cases as abnormal [48]. A study of the Arabic SDQ found that the five-factor structure did not provide a good fit with children ages 6–16, suggesting that certain items may function differently in Arab populations and should be examined further with this population to establish meaningful and relevant constructs [49].

In spite of the growing literature examining the cross-cultural validity of the SDQ, few such studies have been conducted in Latin America. We were able to locate only four published studies. One by Goodman and colleagues examined SDQ findings among predominately African-Brazilian children ages 5–14 in an island community in Northeast Brazil [50]. This study did not examine measurement invariance or SDQ factor structure, but the findings suggested that some subscales may have been overreported given the low impact attributed by parents. A recent study by Gaete et al. [51] assessing the construct validity and reliability of both the self-reported and parent Spanish language SDQ instruments with Chilean adolescents ages 9–15 and their parents found the original five-factor SDQ structure to perform well. As cited by Gaete et al. [51], two additional studies on the SDQ were conducted in Chile. One study by Caqueo et al. attempted to identify response and other differences between Aymara (indigenous) and non-Aymara Chilean children on the self-reported and parent or teacher-reported Spanish-language SDQ without assessing cross-cultural measurement invariance [52]. The second study by Brown et al. failed to reproduce either the standard five-factor SDQ or other previously reported models [53]. Like Gaete et al. [51], we were unable to find additional studies of the psychometric properties of the SDQ in other Latin American countries.

In summary, existing evidence suggests that the SDQ is highly effective for screening children’s mental health problems in Western European and U.S. contexts, but may be less effective outside of these contexts. The standard five-factor solution may also not translate cross-culturally. Few studies have examined the psychometric properties of the SDQ among Latin American populations [50– 53]. The study reported in this paper is designed to help fill this gap in the literature. The purpose of this study was twofold: (1) examine the factor structure of the parent or teacher-reported Spanish language version of the SDQ for children ages 4–17 with parents or other caregivers of primary school-aged children from the Department of Intibucá in Honduras; and (2) assess the measurement invariance of the best fitting SDQ model based on respondent and child gender.

## Materials and methods

### Setting: Department of Intibucá, Honduras

Honduras is a country in the “Northern Triangle” region of Central America that experiences a high rate of poverty (63%) [54]. Honduras also experiences one of the highest rates of violent crime in the world, with 67 homicides per 100,000 inhabitants in 2014 [55], although this rate dropped to 43.6 homicides per 100,000 inhabitants in 2017 [56]. The country is divided into 18 departments and each department is subdivided into municipalities. The Department of Intibucá–the location of this study–is a largely rural area that experienced 29.9 homicides per 100,000 inhabitants in 2017, one of the lower rates in the country [56].

### Data collection/participants

The sampling frame for the study included beneficiaries of the U.S. Department of Agriculture (USDA)-funded Food for Education program, implemented by Catholic Relief Services, who participated in an external evaluation of the program in 2016 led by two of the authors (TMC and JA). Parents or other caregivers (e.g., grandmothers, aunts, uncles, and siblings) of 1,244 Honduran children completed survey measures in 180 randomly selected schools from a population of 1,047 schools in the Department of Intibucá. All respondents provided verbal informed consent prior to participation. This study was approved by the Boston College Institutional Review Board.

The sample included in this study are parent or other caregiver respondents (*n* = 967) for 477 male and 490 female children from preschool (ages 4–5), primary school (ages 6–11), and secondary school (ages 12+) [57]. Respondent caregivers ranged in age from 16 to 88 (*M =* 35.84, *SD* = 11.99) and were primarily female (*n* = 831). Mothers were reported as children’s main caregivers (*n* = 755), followed by fathers (*n* = 118), then other caregivers (*n* = 94) (e.g., grandparents, great-grandmothers, siblings, aunts, uncles, and other caregivers living with the children). The majority of respondents (*n* = 706) identified as indigenous Lenca, while 261 did not. Like other indigenous groups in the region, the Lenca have suffered oppression and colonization for centuries and continue to experience social marginalization [58], including the loss of their distinct Lenca language [59]. Exclusion criteria for this study included being outside of the age range of 4 to 17, as well as missing all demographic data; because of enumerator error or technological problems in synchronizing electronically collected data, demographic information was not collected for 196 parent or other caregiver respondents, although SDQ data was complete for these 196 individuals.

### Instrument

The Spanish language, single-sided SDQ version for parents or teachers of children ages 4–17 was employed in this study (see www.sdqinfo.com). The SDQ is comprised of 25 items measured on a three-point Likert-type scale (0 = “not true,” 1 = “partly true,” or 2 = “certainly true”), and rated by the respondent over the past three months. Items are clustered within the five subscales with five items each: Emotional Symptoms, Hyperactivity, Peer Problems, Conduct Problems, and Prosocial Behavior. The Total Difficulties scale (ranging from 0–40) measures overall functioning as a sum of all subscales except Prosocial Behavior (ranging from 0–10). High Total Difficulties scores indicate higher psychiatric difficulties, while higher Prosocial Behavior subscale scores indicate better functioning [7]. In previous research with British samples, the SDQ has shown good Cronbach’s alpha internal consistency (α = 0.73) and good mean retest stability (α = 0.62) [7], as well as good predictive validity [9].

The SDQ was administered alongside surveys on parents’ perceptions of Food for Education program operations and effectiveness. Respondents completed the SDQ in this context given the known links between children’s emotional and behavioral problems and academic performance [60], and that schoolchildren in low- and middle-income countries are often at increased risk for psychosocial problems [61]. In surveys, parents and other caregivers were also asked demographic questions, including the date of birth of the child, respondent gender, child gender, the child’s primary caregiver, municipality in which they lived within the Department of Intibucá, and whether they considered themselves to be members of the indigenous Lenca group.

### Data analysis

SDQ data were complete. Analyses were conducted using Mplus Version 7.4 [62] on the Boston College Linux cluster and in R version 3.4.4 [63]. Descriptive statistics reported include univariate and bivariate statistics, as well as polychoric correlations and internal consistency reliability indexes for categorical data. We randomly split our sample into two groups, one in which we conducted EFA, the other in which we confirmed the best fitting EFA model with CFA. Others have performed split sample EFA and CFA analyses when unable to find an acceptable fitting previously identified SDQ model [35, 47]. Ordered categorical data have thresholds rather than intercepts. As such, we applied the recommended weighted least squares means and variance adjusted (WLSMV) estimation for categorical data along with theta parameterization in EFA and CFA [64, 65].

#### Internal consistency reliability

As a discrimination index, we present the item-rest correlations for SDQ items based on ordinal alpha (α) [66] for the full sample and male and female respondent and child genders. However, to assess the internal consistency reliability of the standard five-factor SDQ solution, we followed Stone et al. by presenting McDonald’s ω for each factor individually [29, 30], as well as ordinal α [66] like Ortuño-Sierra et al. [34, 35, 39]. We chose to report McDonald’s ω and ordinal α over similar indexes, such as Raykov’s composite reliability for congeneric measures index [67, 68], both to allow for comparison with Stone et al. [29] and Ortuño-Sierra et al. [39], as well as because Widhiarso and Ravand caution against the use of Raykov’s index with categorical data with limited response options, instead recommending the use of indexes that support WLSMV estimation for categorical data [69]. We calculated both ordinal α and McDonald’s ω in R using syntax provided by Gadermann and colleagues [70] and the R “psych” package [71].

#### Exploratory factor analysis

We employed oblique geomin rotation in Mplus allowing for correlations between factors for EFA [65]. Factor loadings > 0.300 were retained [72]. We assessed EFA model fit using comparative fit index (CFI) and Tucker-Lewis Index (TLI) close to 1.00, but at least > 0.90, root mean square error of approximation (RMSEA) < 0.05 or at most < 0.08, and standardized root mean square residual (SRMR) < 0.08 or at most < 0.10 [73, 74]. We also considered higher communalities (*h*2) desirable (> 0.600), which represent the amount of an item’s shared factor variance [75, 76], as well as eigenvalues > 1.00, which represent the amount of variance explained by a factor solution [76]. Parallel analysis is also employed by some researchers for determining factor solutions. However, it is not available for categorical data using WLSMV estimation in Mplus due to inadequate performance [77]. Instead, we employed the R package “random.polychor.pa”, which performs parallel analysis with polychoric correlations for ordered categorical data, as well the Velicer minimum average partial (MAP) (4th power) method [78].

#### Confirmatory factor analysis

In addition to CFI, TLI, and RMSEA fit values employed in EFA, a CFA model may be considered to have a good fit if the weighted root mean square residual (WRMR) is around 1.00 [79], although the WRMR fit index is considered experimental [80]. However, Kenny noted that in CFA, the commonly used incremental fit indexes CFI and TLI relate to the degree of correlation among scale variables, where low levels of average correlations between variables can be associated with low CFI and TLI [81]. Kenny further noted that when a model’s RMSEA is 0.05 and TLI is 0.90, null (or baseline in Mplus) model RMSEA is 0.158 [81]. Moreover, Kenny stated “this mathematical fact that a model whose null model RMSEA is less than 0.158 and whose RMSEA is 0.05 must have a TLI of less than .90 [sic] is something that has never been published but is in fact true”, leading to the recommendation that researchers should determine null model RMSEA and if < 0.158, indexes like CFI and TLI may not be useful in assessing model fit [81]. As described by Gomez and Stavropoulos [46], previous studies that showed low CFI and TLI values and acceptable RMSEA for the SDQ may be due to low average correlations between SDQ items, suggesting that these studies actually showed good fits for the five-factor SDQ if RMSEA had been considered as the primary fit index rather than CFI or TLI. In this paper, we first assessed baseline model RMSEA before determining which goodness of fit index to report for CFA. Because Mplus does not calculate baseline model RMSEA automatically, we followed Kenny and calculated it as follows, where χ^2^ represents chi-square, *df* the degrees of freedom, and *N* the sample size of each null, or baseline, model [81]:

$$\mathrm{RMSEA}=\frac{\sqrt{\left(\chi^{2}-df\right)}}{\sqrt{\left[df\left(N-1\right)\right]}}$$ (1)

Baseline RMSEA was 0.106 for the full sample and < 0.158 for the subsamples [81], suggesting we should use RMSEA rather than CFI or TLI when considering CFA model fit.

While a non-significant χ^2^ is suggestive of a good fit, this statistic is sensitive to sample size [82]. Other goodness of fit statistics, if showing an acceptable fit, can be referred to in place of χ^2^ [83]. When a model was not identified, we iteratively altered single loadings like Bøe et al. [23]. After identifying the best fitting model, we assessed that model for measurement invariance based on caregiver respondent and child gender, including pursuing partial invariance as needed [82].

#### Measurement invariance testing

The best fitting split sample CFA model was assessed for measurement invariance for caregiver respondent gender (male or female), and child gender (boy or girl). Measurement invariance tested typically involves tested nested hypotheses: H1) configural invariance (equal factor structure); H2) metric invariance (equal factor loadings); H3) scalar invariance (equal item thresholds); and H4) strict invariance (equal item residuals) [73]. However, because strict invariance is overly restrictive in real world practice, findings of configural, metric, and scalar invariance are acceptable [73]. Furthermore, if items are cross-loaded on more than one factor, metric invariance is not available for categorical data in Mplus; rather comparisons should be made using the scalar invariance model [84]. We employed Mplus chi-square difference testing (χ^2^_diff_) when comparing nested CFA models, as chi-square difference is not distributed as chi-square in WLSMV estimation [65].

## Results

### Descriptive statistics and correlations

Compared to normative data (based on a sample of UK children) [7], the current sample is at the high-normal range for Total Difficulties (*M* = 12.78, *SD* = 5.99); borderline range for Emotional Symptoms (*M* = 3.87, *SD* = 2.80); high-normal range for Conduct Problems (*M* = 1.97, *SD* = 1.87); normal range for Hyperactivity (*M* = 4.27, *SD* = 2.22); borderline range for Peer Problems (*M* = 2.65, *SD* = 1.88); and normal range for Prosocial Behavior (*M* = 7.93, *M* = 1.91). While no comparative data for Honduras are available, Goodman et al. presented SDQ data collected from a rural setting in Brazil with a sample of relatively poor parents [50]. Compared to these data, the Honduran parents and other caregivers from Intibucá in the current study showed lower Total Difficulties, Emotional Symptoms, and Conduct Problems scores; comparable scores for Hyperactivity; higher scores for Peer Problems; and lower scores for Prosocial Behavior [50]. Some differences were also seen between the Honduran respondents and the Chilean parent-reported data presented by Gaete et al. [51]. Specifically, Honduran parents and other caregivers had higher mean Emotional Symptoms, Hyperactivity, and Peer Problems scores, comparable mean Conduct Problems scores, and lower mean Prosocial Behavior scores [51]. Polychoric correlations between items were acceptable for the full sample (Table 1). The Kaiser-Meyer-Olkin (KMO) test was quite close to good (> = 0.80) at 0.796, and a statistically significant Bartlett’s test of sphericity showed that factor analysis was appropriate for this correlation matrix: χ^2^ = 2,465.77 (*df* = 300), *p* < 0.001 [76].

**Table 1:** Parent or Teacher Spanish Language SDQ Polychoric Correlation Matrix (n = 967).

Item	1	2	3	4	5	6	7	8	9	10	11	12	13	14	15	16	17	18	19	20	21	22	23	24	25
**1**	1.00																								
**2**	.05	1.00																							
**3**	-.04	.06	1.00																						
**4**	.19	.06	-.09	1.00																					
**5**	.21	.18	.11	.01	1.00																				
**6**	-.15	.06	.20	-.18	-.04	1.00																			
**7**	.08	.12	-.04	-.09	.26	.02	1.00																		
**8**	.08	.13	.33	.03	.21	.20	.09	1.00																	
**9**	.30	-.04	.01	.21	.03	-.09	-.16	.02	1.00																
**10**	.11	.41	.07	.09	.31	.04	.31	.15	.04	1.00															
**11**	-.09	-.03	.14	-.33	.05	.13	.30	.06	-.17	-.01	1.00														
**12**	.01	.31	.22	.00	.34	.16	.26	.29	-.06	.43	.15	1.00													
**13**	.01	.12	.40	-.03	.26	.23	.15	.43	-.07	.17	.16	.32	1.00												
**14**	-.17	.00	.13	-.20	.10	.07	.20	.03	-.18	.04	.38	.12	.12	1.00											
**15**	.01	.23	.19	.07	.15	.17	.02	.20	.02	.26	-.08	.19	.13	-.12	1.00										
**16**	.08	.11	.37	.06	.19	.14	.11	.38	.04	.17	.12	.26	.43	.05	.21	1.00									
**17**	.01	-.12	-.06	.04	-.28	.00	-.39	-.08	.16	-.25	-.16	-.37	-.17	-.36	-.08	-.17	1.00								
**18**	.10	.13	.19	.03	.29	.13	.30	.26	-.06	.21	.19	.40	.33	.15	.24	.33	-.25	1.00							
**19**	.06	.13	.29	.01	.23	.15	.18	.37	-.10	.20	.19	.39	.41	.17	.21	.35	-.14	.30	1.00						
**20**	.16	-.02	-.03	.16	-.06	-.09	-.22	.12	.39	-.01	-.28	-.11	-.10	-.22	.05	.03	.23	-.10	.02	1.00					
**21**	.07	.20	-.06	-.06	.22	-.11	.30	-.01	-.16	.16	.21	.16	.08	.19	.04	.15	-.28	.14	.09	-.23	1.00				
**22**	.03	.11	.16	.00	.24	.14	.35	.16	-.17	.31	.07	.43	.30	.27	.17	.12	-.29	.33	.33	-.11	.11	1.00			
**23**	-.14	.07	.14	-.10	-.05	.31	.07	.17	-.07	.02	.06	.07	.18	.08	.09	.12	-.03	.01	.12	.02	-.16	.19	1.00		
**24**	.06	.04	.33	.11	.13	.17	.05	.35	-.07	.09	.03	.26	.43	.09	.24	.52	-.09	.20	.34	.00	.01	.25	.04	1.00	
**25**	.10	.17	.18	-.06	.20	-.05	.36	.10	-.21	.11	.18	.20	.23	.15	.11	.27	-.34	.28	.27	-.11	.39	.35	-.09	.16	1.00

Rounded to the nearest hundredth place.

### Standard 5-Factor SDQ internal consistency reliability

Item-rest correlations for SDQ items, factor and scale ordinal α, and McDonald’s ω are presented in Table 2 for children and respondents by gender, along with factor means and standard deviations for each group. Item-rest correlations were low for all four groups. Individual factor internal consistency reliability expressed as McDonald’s ω for the standard five-factor SDQ solution ranged from poor to good for female and male respondents and for girls and from poor to acceptable for boys. Ordinal α ranged from poor to acceptable for all four subgroups. The Total Difficulties score, which included all items aside from the Prosocial Behavior factor, was good for all subgroups based on both McDonald’s ω and ordinal α. These findings were similar to the full Honduran sample (Total Difficulties ω = 0.84, Emotional Symptoms ω = 0.79, Conduct Problems ω = 0.74, Hyperactivity ω = 0.67, Peer Problems ω = 0.59, Prosocial Behavior ω = 0.59) (not shown in Table 2). Ordinal α was lower than McDonald’s ω for all factors for each subgroup shown in Table 2, the same as with the full Honduran sample (Total Difficulties α = 0.81, Emotional Symptoms α = 0.77, Conduct Problems α = 0.70, Hyperactivity α = 0.57, Peer Problems α = 0.50, Prosocial Behavior α = 0.53) (not shown in Table 2). Together these findings suggest that some factors in the standard five-factor solution may not be a good fit for this sample of Honduran parents and other caregivers.

**Table 2:** Parent or Teacher Spanish Language SDQ Item-Rest Correlations, Internal Consistency Reliability Indexes, and Mean Scores by Child (Boys n = 490, Girls n = 477) and Respondent (Female n = 831, Male n = 136) Gender.

	Child Gender		Respondent Gender	
SDQ Items & Factors (English Translation)	Boys	Girls	Females	Males
EMOTIONAL SYMPTOMS				
3. Often complains of headaches, stomach-aches or sickness	.41	.53	.47	.47
8. Many worries or often seems worried	.50	.50	.51	.43
13. Often unhappy, depressed or tearful	.57	.58	.59	.44
16. Nervous in new situations, easily loses confidence	.52	.64	.58	.57
24. Many fears, easily scared	.54	.57	.56	.50
Ordinal α	.74	.79	.77	.72
ω	.78	.82	.80	.77
*M* (*SD*)	3.79 (2.74)	3.96 (2.86)	3.76 (2.61)	3.89 (2.83)
CONDUCT PROBLEMS				
5. Often loses temper	.34	.44	.39	.37
7. Generally well behaved, usually does what adults request (R)	.43	.41	.39	.48
12. Often fights with other youths or bullies them	.47	.56	.52	.47
18. Often lies or cheats	.46	.50	.47	.50
22. Steals from home, school or elsewhere	.54	.45	.44	.68
Ordinal α	.69	.71	.69	.74
ω	.74	.79	.73	.84
*M* (*SD*)	2.02 (1.84)	1.92 (1.90)	1.62 (1.74)	2.03 (1.88)
HYPERACTIVITY				
2. Restless, overactive, cannot stay still for long	.45	.37	.43	.26
10. Constantly fidgeting or squirming	.44	.30	.39	.31
15. Easily distracted, concentration wanders	.25	.24	.26	.17
21. Thinks things out before acting (R)	.33	.27	.32	.19
25. Good attention span, sees work through to the end (R)	.26	.34	.30	.31
Ordinal α	.59	.54	.58	.47
ω	.67	.67	.68	.60
*M* (*SD*)	4.45 (2.23)	4.12 (2.20)	4.01 (2.08)	4.33 (2.24)
PEER PROBLEMS				
6. Would rather be alone than with other youth	.30	.23	.27	.27
11. Has at least one good friend (R)	.38	.26	.30	.35
14. Generally liked by other youth (R)	.35	.22	.29	.30
19. Picked on or bullied by other youth	.29	.22	.27	.17
23. Gets along better with adults than with other youth	.23	.25	.25	.13
Ordinal α	.54	.45	.51	.46
ω	.67	.54	.59	.58
*M* (*SD*)	2.60 (1.90)	2.71 (1.86)	2.51 (1.77)	2.68 (1.90)
TOTAL DIFFICULTIES				
Ordinal α	.80	.82	.81	.81
ω	.84	.85	.85	.86
*M* (*SD*)	12.86 (5.88)	12.71 (6.11)	11.90 (5.63)	12.93 (6.04)
PROSOCIAL BEHAVIOR				
1. Considerate of other people’s feelings	.24	.28	.24	.38
4. Shares readily with other youth, for example books, games, food	.23	.24	.21	.40
9. Helpful if someone is hurt, upset or feeling ill	.41	.49	.46	.39
17. Kind to younger children	.13	.22	.16	.28
20. Often offers to help out (parents, teachers, children)	.44	.33	.38	.41
Ordinal α	.52	.55	.52	.62
ω	.61	.62	.59	.69
*M* (*SD*)	7.87 (1.92)	7.99 (1.91)	7.94 (1.98)	7.93 (1.90)

R = Reverse-coded. α = alpha. ω = McDonald’s omega. *M* = Mean. *SD* = Standard deviation. Item-rest correlations presented based on item “r.drop” in “psych” package [71]

Comparative internal consistency reliability from other studies using the Spanish language SDQ are available. Aside from the Emotional Symptoms subscale that also had an ordinal α of 0.76, Ortuño-Sierra and colleagues [34] reported higher ordinal α values for four SDQ subscales and the Total Difficulties score in a Spanish population than was seen here for the full Honduran sample. The Emotional Symptoms and Conduct subscales and Total Difficulties score ordinal α was comparable to those in a sample of Spanish adolescents [35], although other subscales were lower in the Honduran sample presented here. Furthermore, while the Emotional Symptoms subscale and Total Difficulties score were again comparable with a sample of adolescents from Spain, Conduct Problems ordinal α was slightly lower (0.68) in that population, while other subscales were lower in the Honduran sample [39]. Regarding comparison of McDonald’s ω, all five SDQ subfactors had lower levels of internal consistency reliability in the Honduran sample than reported by Stone with a Dutch sample [29]. These findings suggest the standard five-factor SDQ does not fit the Honduran sample well.

### Split sample exploratory and confirmatory factor analyses

**Fig 1:**
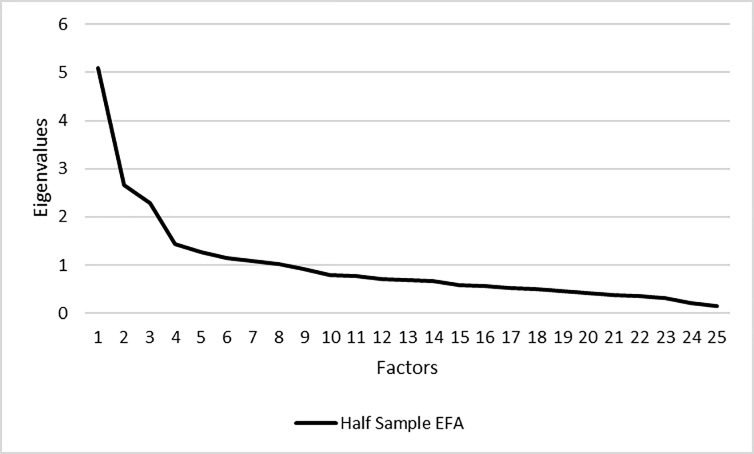
Eigenvalues. WLSMV EFA eigenvalues for the parent or teacher SDQ (Spanish language version) with half of the Honduran parent or other caregiver respondents (*n* = 484).

**Fig 2:**
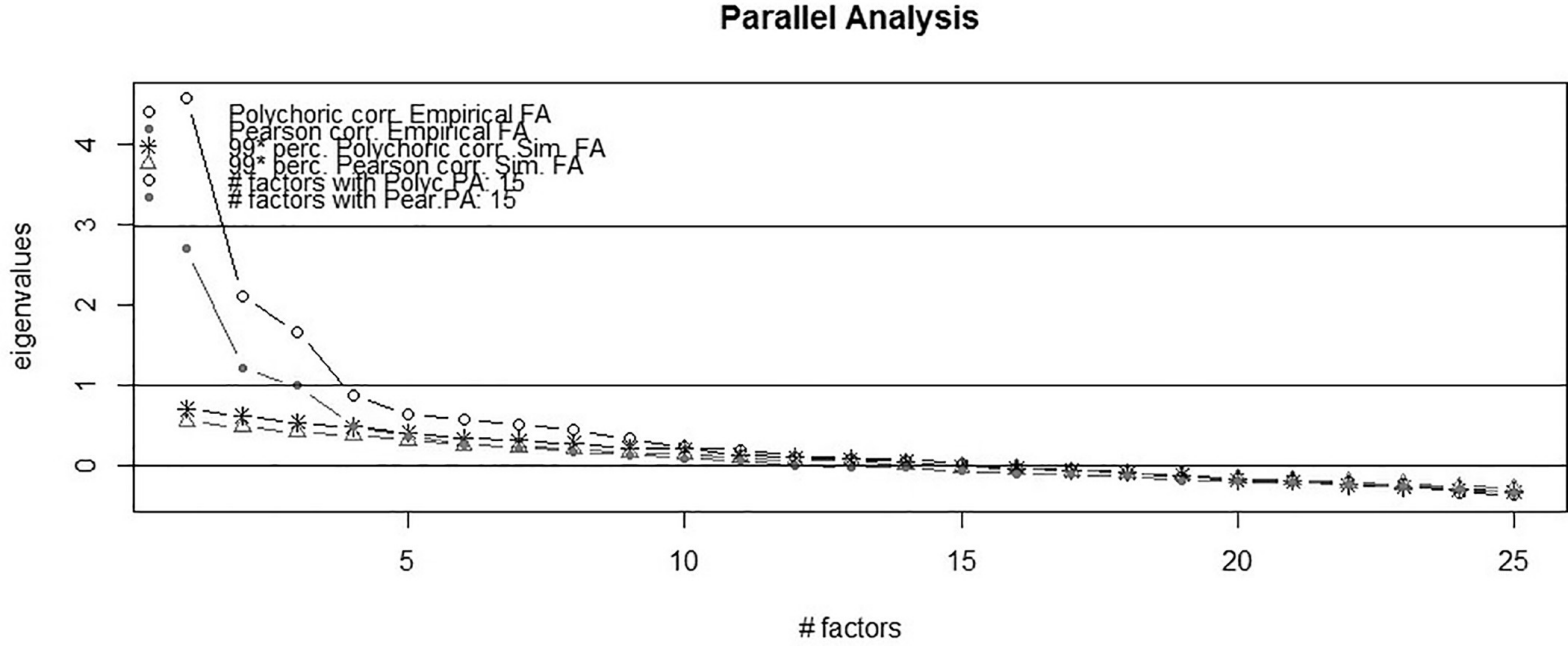
Parallel analysis. Results for the parent or teacher SDQ (Spanish language version) with half of the Honduran parent or other caregiver respondents (*n* = 484). FA = Factor analysis; PA = Parallel analysis.

While we tested a number of SDQ factor models previously identified in the literature (see S1 Table and S1 Fig), including a best-fitting five factor model with reverse-coded items cross-loading on the Positive Behavior factor and five correlating residuals (see S2 Table), we were unable to find a model that allowed for successful measurement invariance testing between groups (see S2 Table notes). Consequently, we randomly split the Honduran sample into two groups, conducting EFA with the one group (*n* = 484) and CFA with the other (*n* = 483). Fig 1 illustrates the eigenvalues generated for the 25 SDQ items for the EFA random sample of Honduran respondents, showing models between one and nine factors having eigenvalues > 1.000. However, parallel analysis with polychoric correlations showed three factors were retained if using the Velicer MAP (4th power) method and 15 factors with parallel analysis (see Fig 2). Yet the EFA model with 15 factors failed to converge in Mplus (not shown).

Extracted EFA model goodness of fit indexes are presented in Table 3. One- and two-factor solutions had poor fits based on CFI and TLI both < 0.90, although RMSEA was acceptable for both models and SRMR for the two-factor model. Model fit for the three-factor solution was acceptable, while the four-factor model showed a poor fit. However, EFA models with five- or more factors showed a better fit, including a non-significant chi-square. Also, models with six- or seven-factors did not differ significantly based on Mplus built-in model difference testing, nor did models with more than seven factors. Because the seven- and eight-factor models do not differ significantly, parsimony supports the seven-factor model over the eight-factor model, which is not presented here.

**Table 3:** EFA Goodness of Fit Indexes for the Parent or Teacher Spanish Language SDQ for Half of the Honduran Respondents (n = 484) .

EFA Extracted Models	χ^2^ (*df*)	χ^2^_diff_ (*df*)	RMSEA (CI)	CFI	TLI	SRMR
1-Factor	751.39 (275)***		.060 (.055-.065)	.67	.66	.114
2-Factor	540.14 (251)***	166.87 (24)***	.049 (.043-.054)	.81	.77	.086
3-Factor	321.19 (228)***	159.05 (23)***	.029 (.021-.036)	.94	.92	.060
4-Factor	244.12 (206)*	70.26 (22)***	.020 (.006-.029)	.98	.96	.052
5-Factor	197.45 (185)^ns^	44.11 (21)**	.012 (.000-.024)	.99	.99	.047
6-Factor	164.02 (165)^ns^	32.48 (20)*	.000 (.000-.020)	1.00	1.00	.042
7-Factor	134.61 (146)^ns^	28.19 (19)^ns^	.000 (.000-.016)	1.00	1.02	.037

*df* = Degrees of freedom.

^ns^ = Not significant. CI = Confidence interval.

* *p* < .05

** *p* < .01

*** *p* < .001.

WLSMV estimation. Rounded to nearest thousandth place. Extraction method: Principal Axis Factoring with geomin oblique rotation. Acceptable fits include non-significant (*p* > .05) χ^2^, RMSEA < .05 or at most < .08, CFI and TLI > .90 and preferably > .95, and SRMR < .10, but preferably < .08 [73, 74].

Table 4 presents Geomin rotated loadings for three to seven-factor extracted models. All models had some items with communalities < 0.600. Emotional Symptoms was the only SDQ subscale consistently reproduced, although the Conduct Problems subscale was reproduced aside for in the seven-factor model, where item 12 “Fights” loaded with three items from the Hyperactivity subscale. Prosocial Behavior was also reproduced aside from one item in three-, four-, and five-factor models, and two items in the six- and seven-factor models. Hyperactivity was reproduced aside from item 15 “Distracted” in the three-factor model, an item that loaded < 0.300 on all three factors. While the seven-factor model showed the most variation (see Table 4), it also had a better fit than models with fewer factors (see Table 3). However, all but one item on the seventh factor, item 23 “Adult best”, loaded to a higher degree on other factors. Together, these findings give weak support for a substantive seventh factor.

**Table 4:** Three-, Four-, Five-, Six-, and Seven-Factor EFA Results for the Parent or Teacher Spanish Language SDQ for Half of the Honduran Respondents (n = 484).

SDQ Items	3-Factor Model				4-Factor Model					5-Factor Model						6-Factor Model							7-Factor Model								
	1	2	3	*h*^*2*^	1	2	3	4	*h*^*2*^	1	2	3	4	5	*h*^*2*^	1	2	3	4	5	6	*h*^*2*^	1	2	3	4	5	6	7	*h*^*2*^	
EMOTIONAL SYMPTOMS																															
3.			.**56**	.69			**.53**		.69				**.52**		.69				**.52**			.64				.**54**				.65	
8.			**.61**	.61			**.54**		.60				**.48**		.60				**.48**			.58				**.44**			.30	.58	
13.			**.64**	.49			**.67**		.49			.31	**.58**		.46			.36	**.58**			.46				**.63**				.45	
16.			**.63**	.51			**.77**		.46				**.67**		.49				**.72**			.38				**.68**			.37	.36	
24.			**.56**	.67	-.33		**.78**		.51				**.83**	-.33	.36				**.79**			.43				**.80**				.36	
CONDUCT PROBLEMS																														
5.		**.41**		.75					.76		.32	**.51**			.69		.34	.**49**				.65	.40	.38						.61	
7.R		**.64**		.60		.**55**			.60			**.67**			.56			.38		.**52**		.48	.**82**							.44	
12.		**.55**		.55	.**41**				.53			**.44**			.53	.33		.**52**				.47			.**43**					.47	
18.		**.50**		.67					.67			**.52**			.65			.35				.64	.**50**							.62	
22.		**.58**		.59		.**43**			.60			**.56**			.59			.35		.38		.57	.**65**							.53	
HYPERACTIVITY																															
2.		.36		.80	**.65**				.62	**.77**					.42	**.72**						.41			**.70**					.46	
10.		**.51**		.63	**.65**				.52	**.50**		.31			.56	**.53**		.33				.51			**.60**					.50	
15.				.84					.83	.35					.79	.32						.80	.32		.35		**-.46**	-.**52**	.37	.65	
21.R		**.56**		.70		**.42**		.40	.65			.38		-.**43**	.62					**.42**	-.35	.62								.57	
25.R		.**56**		.69		**.43**		.31	.62			.**41**		-.38	.61					**.57**		.52	.35					-.**41**		.49	
PEER PROBLEMS																															
6.	-.32		.**46**	.72				-.**50**	.67		-.35				.68		-.31					.68						.**43**		.65	
11.R	**-.45**			.73		**.49**			.73			.37			.71			**.41**				.69					.**41**			.69	
14.R	**-.44**	.**44**		.64		**.56**			.64		-.32	**.45**			.64			**.45**				.63					**.57**			.53	
19.		.32	**.40**	.66			.49		.66			.35	.40		.65			.31	**.41**			.66				.**46**				.63	
23.			.36	.84				-.**52**	.72					**.50**	.66						**.68**	.48							.34	.54	
PROSOCIAL BEHAVIOR																															
1.	**.43**			.81				.34	.81		**.55**				.72		**.53**					.73		**.52**						.72	
4.	.**59**			.66		**-.41**		.**44**	.63		**.52**				.64		**.50**					.65		**.41**			-.39			.63	
9.	**.52**			.68		**-.54**			.68		**.62**				.54		**.61**					.54		**.61**						.53	
17.		**-.68**		.52		**-.56**			.51			-.**65**			.51			-.**48**		-.35		.50	-.**60**							.50	
20.	.40	-.34		.74		**-.54**			.72		.31	-.31			.72				-.31			.71	-.**42**					.**56**	.31	.65	

*h*^*2*^=Item commonality, variance reproduced. R = Reverse-coded. WLSMV estimation. Rounded to nearest hundredth place. Only factor loadings over .300 are presented. Factor loadings over .400, the preferred minimum [82], are bolded. Extraction method: Principal Axis Factoring with geomin oblique rotation.

Based on the Velicer MAP (4th power) method of parallel analysis showing we should retain three factors, we tested the three-factor model in the second random sample using CFA, which dropped item 15 “Distracted” and allowed items 6 “Loner”, 14 “Popular”, 19 “Bullied”, and 20 “Help out” to cross-load on multiple factors. However, we found that while RMSEA was acceptable at 0.040, CFI and TLI were < 0.90 in CFA, suggesting a poor fit for the three-factor model compared to the EFA sample (see Table 5). This finding suggests that following Kenny [81] and Gomez and Stavropoulos [46] in first assessing baseline RMSEA for determining which fit index to use may not produce a good fitting model. We then tested four- to seven-factor models using CFA in the second sample in an attempt to find a better fitting model, also presented in Table 5. However, the six-factor model would not converge, even when starting values equal to half the item variance were added. Only the seven-factor model approached an acceptable fit with CFI = 0.90. In the spirit of model parsimony, we selected the three-factor model for measurement invariance testing. SDQ subscale items also loaded together in the three-factor model more so than in other extracted models, where Factor 1 appears to expand the Prosocial Behavior factor by including negatively loading items 6 “Loner”, 11 “Friend”, and 14 “Popular” related to Peer Problems. This suggests that not being a loner, having at least one good friend, and generally being liked by other youth are related to Prosocial Behaviors in this sample. Factor 2 includes other previously identified three-factor model’s Externalizing factor composed of Conduct Problems and Hyperactivity subscales, along with Peer Problems items 14 “Popular” and 19 “Bullied”, as well as negatively loaded Prosocial Behavior items 17 “Kind” and 20 “Help out”. This suggests that conduct problems and hyperactivity are associated with less reported popularity, being bullied, as well as less reported kindness to younger children or helping others out. Finally, Factor 3 mostly replicates the Internalizing factor from other three-factor models by including all Emotional Symptoms subscale items, along with three items from the Peer Problems subscale, specifically items 6 “Loner”, 19 “Bullied”, and 23 “Adult best”.

**Table 5:** Second Split Sample CFA (n = 483) with the Parent or Teacher Spanish Language SDQ.

CFI Models	χ^2^ (*df*)	RMSEA (CI)	CFI	TLI	WRMR
3-Factor	437.68 (245)***	.040 (.034-.046)	.87	.85	1.17
4-Factor	683.15 (268)***	.057 (.051-.062)	.68	.64	1.53
5-Factor	461.56 (256)***	.041 (.035-.047)	.86	.84	1.15
6-Factor^a^	No convergence.				
7-Factor	390.99 (245)***	.035 (.028-.042)	.90	.88	1.03

*df* = Degrees of freedom. CI = Confidence interval.

*** *p* < .001. WLSMV estimation. Rounded to nearest thousandth place. Acceptable fits include non-significant χ^2^ (*p* > .05), RMSEA < .05 or at most < .08, CFI and TLI > .90 and preferably > .95, and WRMR around 1.00 [73, 74, 79].

^a^Outcome both with and without starting values equal to half the item variance.

### Measurement invariance testing of EFA-extracted 3-factor model

#### Respondent gender

The three-factor model required starting values equal to have the item variances in order to run for male respondents: χ^2^ (*df* = 245, *n* = 136) = 309.62, *p* = 0.003, RMSEA = 0.044 (CI 0.027–0.058), CFI = 0.81, TLI = 0.78, WRMR = 0.995. The model had a better fit with female respondents, but CFI was still < 0.90: χ^2^ (*df* = 245, *n* = 831) = 607.03, *p* < 0.001, RMSEA = 0.040 (CI 0.037–0.045), CFI = 0.87, TLI = 0.85, WRMR = 1.38. Neither configural or scalar invariance models would converge for the three-factor model between respondent genders due to problems with factor one in the male respondent group, suggesting the model varied between respondent genders. This finding also further supported relying on CFI and TLI rather than RMSEA when assessing model fit regardless of baseline RMSEA. Sample size of male respondents may also have been inadequate given the number of items in the SDQ scale.

#### Child gender

**Fig 3:**
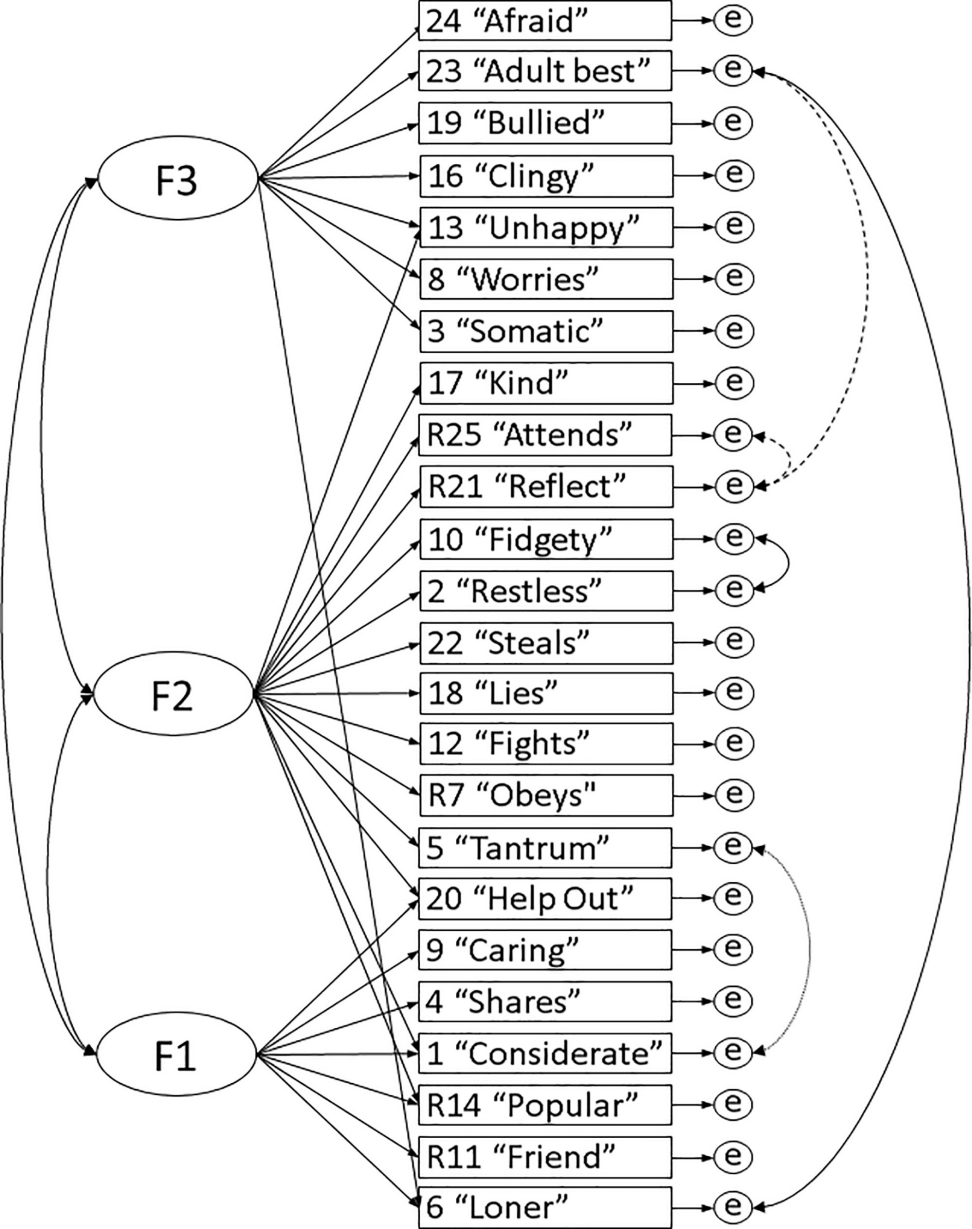
Modified EFA-Extracted three-factor model for child gender. Solid item residual correlations are for both genders. Dashed item residual correlations are for girls. Dotted item residual correlations are for boys.

The three-factor model approached an acceptable fit (CFI < 0.90) for boys (χ^2^ [*df* = 245, *n* = 490] = 434.98, *p* < 0.001, RMSEA = 0.040 [CI 0.034–0.046], CFI = 0.86, TLI = 0.84, WRMR = 1.17) and girls children separately (χ^2^ [*df* = 245, *n* = 477] = 426.72, *p* < 0.001, RMSEA = 0.039 [CI 0.033–0.046], CFI = 0.88, TLI = 0.87, WRMR = 1.15). The three-factor model also showed both configural (χ^2^ [*df* = 490, *n* = 967] = 861.75, *p* < 0.001, RMSEA = 0.040 [CI 0.035–0.044], CFI = 0.87, TLI = 0.85, WRMR = 1.64) and scalar invariance (χ^2^ [*df* = 536, *n* = 967] = 893.72, *p* < 0.001, RMSEA = 0.037 [CI 0.033–0.041], CFI = 0.88, TLI = 0.87, WRMR = 1.69) between child genders based on no significant difference between nested configural and scalar models: χ^2^ (*df* = 46, *n* = 967) = 51.95, *p* = 0.253. However, model fit was not acceptable with CFI < 0.90, even though RMSEA was < 0.05. To improve fit, we allowed up to five correlating residuals based on the greatest reduction in chi-square for each group (four for girls, three for boys, see Fig 3), giving an acceptable fit based on CFI and TLI for girls (χ^2^ [*df* = 241, *n* = 477] = 364.83, *p* < 0.001, RMSEA = 0.033 [CI 0.026–0.039], CFI = 0.92, TLI = 0.91, WRMR = 1.04) and approaching an acceptable fit for boys (χ^2^ [*df* = 242, *n* = 490] = 378.19, *p* < 0.001, RMSEA = 0.034 [CI 0.027–0.040], CFI = 0.90, TLI = 0.88, WRMR = 1.07). We then pursued partial measurement invariance. The modified three-factor model showed both partial configural (χ^2^ [*df* = 483, *n* = 967] = 743.08, *p* < 0.001, RMSEA = 0.033 [CI 0.029–0.038], CFI = 0.91, TLI = 0.90, WRMR = 1.49) and scalar invariance (χ^2^ [*df* = 529, *n* = 967] = 782.58, *p* < 0.001, RMSEA = 0.031 [CI 0.027–0.036], CFI = 0.91, TLI = 0.91, WRMR = 1.56), with acceptable CFI > 0.90 and no significant difference between nested models: χ^2^ (*df* = 46, *n* = 967) = 53.76, *p* = 0.203.

### Internal consistency reliability for the EFA-Extracted 3-factor model

When internal consistency reliability was assessed for the EFA-extracted 3-factor model for the full sample (*n* = 967), ordinal α was 0.63 for factor one, 0.77 for factor two, and 0.76 for factor three. Like the standard 5-factor SDQ model, McDonald’s ω higher at 0.71 for factor 1, 0.81 for factor 2, and 0.83 for factor 3. As shown in Table 6, similar poor to acceptable levels of factor ordinal α and acceptable to good levels of McDonald’s ω were seen for each factor for child and respondent genders. However, factor one had generally low item-rest correlations, as did a number of items that cross-loaded on multiple factors, such as items like 6 “Loner” and 14 “Popular”, with more items having low item-rest correlations than in the standard five-factor model (see Table 2).

**Table 6:** Parent or Teacher Spanish Language SDQ: EFA Extracted Three-Factor Model Item-Rest Correlations and Internal Consistency Reliability Indexes by Child (Boys n = 490, Girls n = 477) and Respondent (Female n = 831, Male n = 136) Gender.

	Child Gender		Respondent Gender	
Extracted Factors & Abbreviated SDQ Items	Boys	Girls	Females	Males
Factor 1				
6 “Loner”	-.08	-.19	-.17	.07
11 “Friend” (R)	-.08	-.22	-.18	-.01
14 “Popular” (R)	-.14	-.14	-.14	-.09
1 “Considerate”	.12	.08	.09	.18
4 “Shares”	-.05	-.08	-.10	.18
9 “Caring”	.15	.28	.22	.16
20 “Help out”	.09	-.01	.02	.19
Ordinal α	.64	.62	.64	.58
ω	.71	.74	.76	.74
Factor 2				
14 “Popular” (R)	.11	.13	.10	.26
1 “Considerate”	.19	.11	.14	.22
20 “Help out”	-.11	-.16	-.11	-.30
5 “Tantrum”	.45	.40	.42	.46
7 “Obeys” (R)	.41	.32	.34	.48
12 “Fights”	.43	.56	.50	.43
18 “Lies”	.41	.47	.43	.47
22 “Steals”	.46	.48	.46	.48
2 “Restless”	.36	.26	.34	.10
10 “Fidgety”	.40	.41	.41	.40
21 “Reflect” (R)	.26	.30	.28	.28
25 “Attends” (R)	.38	.42	.40	.45
17 “Kind”	-.48	-.44	-.43	-.63
19 “Bullied”	.47	.38	.43	.41
Ordinal α	.77	.77	.76	.80
ω	.82	.81	.81	.86
Factor 3				
6 “Loner”	.34	.28	.32	.27
3 “Somatic”	.41	.54	.48	.41
8 “Worries”	.50	.54	.53	.45
13 “Unhappy”	.61	.58	.60	.54
16 “Clingy”	.49	.60	.54	.54
19 “Bullied”	.48	.47	.48	.44
23 “Adult best”	.18	.30	.25	.17
24 “Afraid”	.48	.54	.52	.44
Ordinal α	.74	.78	.77	.71
ω	.82	.82	.82	.81

*Note*. Drops item 15 “Distracted”. R = Reverse-coded. α = ordinal alpha. ω = McDonald’s omega. *M* = Mean. *SD* = Standard deviation. Item-rest correlations presented based on item “r.drop” in “psych” package [71].

## Discussion

The SDQ parent or teacher version was administered to parents and other caregivers in 180 schools in the predominantly rural Department of Intibucá in Honduras. In summary, after being unsuccessful in identifying a good fitting previously identified SDQ factor model, including the standard five-factor model, we randomly split the sample into two groups, conducting EFA with one group and then confirming the best fitting EFA model with CFA in the second group. Parallel analysis using the Velicer MAP (4th power) method suggested a three-factor model would be a good fit and most parsimonious. The extracted three-factor model was also similar to other three-factor models with Internalizing, Externalizing, and Prosocial Behavior factors [16, 39], but dropped item 15 “Distracted”. However, CFA showed that the extracted three-factor model did not fit the second sample well based on CFI and TLI < 0.90. While EFA models with four- to seven-factors had improved fits, only the seven-factor model approached an acceptable fit (CFI = 0.90) in CFA with the second sample, and the six-factor model would not converge. Yet all but one item in the seven-factor model loaded to a greater extent on other factors, leading to our assessing the EFA extracted three-factor model for measurement invariance by gender with the full sample. CFI was still < 0.90 for both male and female respondents, and configural and scalar measurement invariance models would not converge due to issues with factor one for male respondents. However, we successfully conducted measurement invariance testing between child genders for a modified three-factor model by allowing correlating item residuals for boys and girls, showing that the modified three-factor EFA-extracted model was partially invariant between genders. Nevertheless, this model drops item 15 “Distracted”, suggesting that the SDQ may need further refinement with this population in Honduras.

Unlike the samples studied by Stone et al. [29], we did not find all five SDQ subfactors to have acceptable to very good internal consistency reliability using McDonald’s ω [30]. Rather, factor ω for all Honduran respondents ranged from poor to acceptable, and poor to good for subgroups based on respondent and child gender. The exception was the Total Difficulties score, which showed good internal consistency based on McDonald’s ω for all groups. Factor ordinal α were also all lower than ω. Compared to Ortuño-Sierra and colleagues [34, 35, 39], who employed ordinal α with Spanish populations, only the Emotional Symptoms subscale and Total Difficulties ordinal α were acceptable in our sample. Of note, the Emotional Symptoms subscale was the most consistently reproduced SDQ subscale in all EFA analyses presented here. Also, item 19 “Bullied” tended to load with Emotional Symptoms items. This suggests that Honduran parents and other caregivers may perceive children who are bullied as experiencing emotional symptoms as measured by the SDQ.

Furthermore, aside from acceptable RMSEA following Kenny [81], we did not find the standard five-factor SDQ to be a good fit with the sample of Honduran parents and other caregivers. This finding was more in line with Brown et al. [53], and in contrast to Gaete and colleagues, who did find it fit well [51]. We also found that assessing baseline RMSEA in determining which goodness of fit index to report, CFI/TLI or RMSEA like Kenny [81] and Gomez and Stavropoulos [46] produced models with poor fits that we were unable to assess for measurement invariance (e.g., respondent gender). Consequently, relying on CFI and TLI when assessing CFA model fit may be more useful in determining models for measurement invariance testing regardless of baseline RMSEA.

Future research could assess the cross-cultural measurement invariance of the Spanish language SDQ in other samples of Honduran children and their caregivers. If results continue to show differences in factor structure from the original SDQ, further scale development and translation may be useful. Tran et al. [83] support the use of a modified version of the committee-based translation process suggested by Harkness and referred to as *Translation*, *Review*, *Adjudication*, *Pre-testing*, and *Documentation* (TRAPD) [85]. Specifically, Tran et al. recommend assembling an advisory committee with relevant cultural and linguistic expertise with the research area(s) and population(s) [83]. This stage is followed by conducting forward and backward translation of a scale by bilingual translators with proficiency in both languages, that of the initial scale, and the language of the translated version [83]. Tran et al. then endorse a robust evaluation of the translated version using an array of methods [83]. These comprise “expert appraisal and review (evaluation committee), cognitive interviews, focus groups, and pilot testing” (p. 30), all of which provide data for the advisory committee to evaluate the translation’s language clarity, appropriateness, difficulty, and relevance. As in scale development, pilot testing is a key part of translating a scale cross-culturally [83]. In addition to quantitative methods of assessing the translated scale’s reliability and validity, translation validity can also be enhanced through the use of structured interviews during the pilot testing phase [83]. The finalization of the cross-cultural translation process is the last phase, where the advisory committee reviews the results and settles on a final version of the translated scale with the research team [83].

An alternative includes revising the SDQ to be more independent of cultural constructs in relation to the factors it attempts to measure in order to allow for cross-country comparisons of SDQ scores [41]. Researchers could also develop and test a short-form version of the SDQ that excludes questions that tend to differ between cultures. Even in a short-form version, less culturally-sensitive questions could be tested and added or replace those that perform poorly across cultures.

While titled the “Strengths and Difficulties Questionnaire”, SDQ questions are most heavily focused on difficulties, which makes intuitive sense as it was designed to help identify children who may have behavioral or psychological issues [7]. The SDQ seems most useful as a screening tool. As recommended by Stevanovic et al. [41], psychological evaluations and other clinical measures should be used for diagnostics and monitoring children’s outcomes. Furthermore, if the SDQ continues to perform less than optimally with other Honduran samples and reconfiguration is not attempted, perhaps other validated measures should be used in this population.

### Limitations

This study has limitations. While encompassing 180 schools, data were collected from the Department of Intibucá, one of 18 departments in Honduras. Future studies could evaluate the SDQ more broadly in Honduras. Also, metric measurement invariance testing is necessary for determining if different groups respond similarly to items on a scale, which would allow for comparing differences in responses between groups [86], a critical element of cross-cultural measurement invariance testing. However, Mplus does not produce results for metric invariance models with items cross-loading on more than one factor [84]. Therefore, we did not report metric invariance models here. Rather, lack of significant difference between nested configural and scalar invariance models, the latter of which encompasses metric models, represents measurement invariance between groups. Demographic data were also missing for 196 children, and 97 children were outside the SDQ version age range of 4–17, requiring their exclusion from analyses.

### Conclusions

Culturally relevant tools are needed for child and adolescent mental health screening. Few studies have examined the factor structure of the SDQ, a widely translated psychosocial screening tool, with Latin American populations [50–53]. In this study, we found that the standard five-factor SDQ model was not a good fit for the Spanish language parent or teacher version of the SDQ (for children ages 4–17) with Honduran parents and other caregivers in the Department of Intibucá. After conducting split sample EFA then CFA, we found that an EFA extracted three-factor model dropping item 15 “Easily Distracted” had partial configural and scalar invariance between child genders when allowing select item residuals representing the greatest drop in chi-square to correlate. Measurement invariance models would not converge for respondent gender. The SDQ may benefit from further cross-cultural development and testing in Honduras.

## Supporting information

S1 Fig Baseline SDQ CFA models. E = Emotional Symptoms. C = Conduct Problems. H = Hyperactivity. PE = Peer Problems. PR = Prosocial Behavior. I = Internalizing. EX = Externalizing. D = Difficulties. PO = Positive construal method factor.
(TIF)

S1 Table Confirmatory factor analysis results for previously identified models with the parent or teacher Spanish language SDQ for children ages 4–17 with Honduran respondents (n = 967). (DOCX)

S2 Table Confirmatory factor analysis of the best fitting previously identified model, 1c) 5-Factor + 5 Correlating Residualsa + Cross-loading reverse-coded items on the prosocial behavior factor [32, 39], with the parent or teacher Spanish language SDQ by child and respondent gender. (DOCX)
