# Supplementary figures and images for: Assessing the factor structure of the Spanish language parent Strengths and Difficulties Questionnaire (SDQ) in Honduras
Source: PLoS One. 2019 Mar 28;14(3):e0214394. doi: 10.1371/journal.pone.0214394 (PMC6438563; doi:10.1371/journal.pone.0214394)

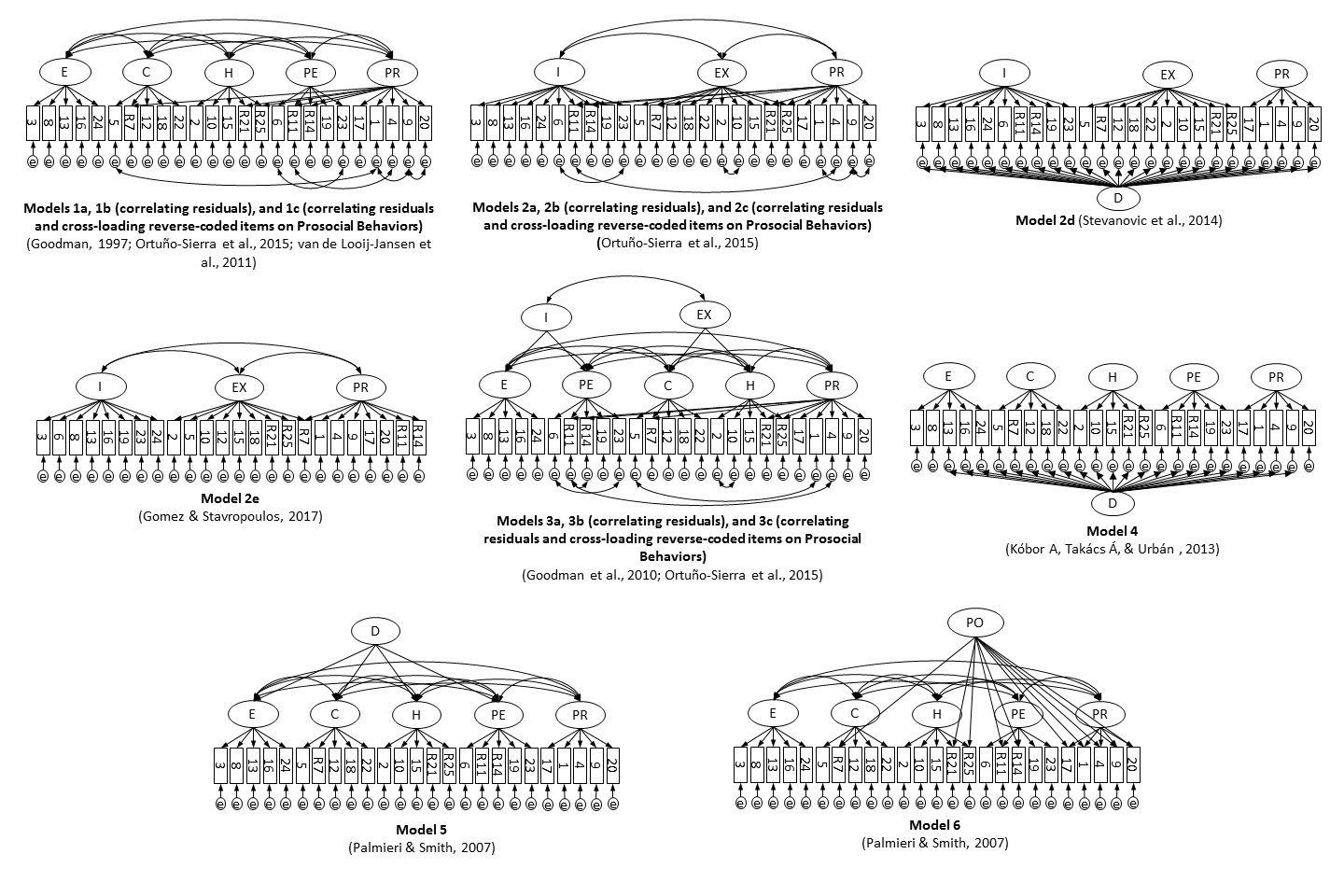

Supplement: S1 Fig — E = Emotional Symptoms. C = Conduct Problems. H = Hyperactivity. PE = Peer Problems. PR = Prosocial Behavior. I = Internalizing. EX = Externalizing. D = Difficulties. PO = Positive construal method factor. (TIF) [file pone.0214394.s001.tif]
