# Supplementary material for: Assessing the factor structure of the Spanish language parent Strengths and Difficulties Questionnaire (SDQ) in Honduras
Source: PLoS One. 2019 Mar 28;14(3):e0214394. doi: 10.1371/journal.pone.0214394 (PMC6438563; doi:10.1371/journal.pone.0214394)
Supplement: S1 Table — (DOCX) [file pone.0214394.s002.docx]

| **S1 Table. Confirmatory Factor Analysis Results for Previously Identified Models with the Parent or Teacher Spanish Language SDQ for Children ages 4-17 with Honduran Respondents (*n* = 967).** | | | | | |
| --- | --- | --- | --- | --- | --- |
| **Models** | **χ^2^ (*df*)** | **RMSEA (CI)** | **CFI** | **TLI** | **WRMR** |
| 1a) 5-factor model [7]^a^ | 882.86 (265)^***^ | .049 (.046-.053) | .81 | .79 | 1.60 |
| 1b) 5-factor + 5 correlating residuals model [34]^a,b^ | 731.48 (260)^***^ | .043 (.040-.047) | .86 | .83 | 1.44 |
| 1c) 5-factor + 5 correlating residuals + cross-loading reverse-coded items on Prosocial Behavior factor model [32, 39]^a,b,c^ | 565.71 (255)^***^ | .035 (.032-.039) | .91 | .89 | 1.25 |
| 2a) 3-factor model [16, 39]^a^ | 937.68 (272)^***^ | .050 (.047-.054) | .80 | .78 | 1.66 |
| 2b) 3-factor + 5 correlating residuals model [39]^a,d^ | 756.21 (267)^***^ | .044 (.040-.047) | .85 | .83 | 1.47 |
| 2c) 3-factor + 5 correlating residuals + cross-loading reverse-coded items on Prosocial Behavior factor model [39]^a,d^ | 595.77 (262)^***^ | .036 (.032-.040) | .90 | .88 | 1.28 |
| 2d) Bifactor model for 3-factor 2a with uncorrelated factors [41] | 696.28 (225)^***^ | .043 (.039-.047) | .86 | .84 | 1.37 |
| 2e) 3-factor slightly reordered from models 2a, 2b, & 2c, drops item 22. Steals + cross-loading reverse-coded items on Prosocial Behavior factor model [16, 46]^a^ | 808.18 (248)^***^ | .048 (.045-.052) | .83 | .81 | 1.59 |
| 3a) 5-factor + second-order Internalizing and Externalizing factors model [12, 39]^e^ | 895.49 (268)^***^ | .049 (.046-.053) | .81 | .79 | 1.62 |
| 3b) 5-factor + second-order Internalizing and Externalizing factors + correlating residuals model [39]^d,e^ | 738.60 (263)^***^ | .043 (.040-.047) | .86 | .84 | 1.45 |
| 3c) 5-factor + second-order Internalizing and Externalizing factors + 5 correlating residuals + cross-loading reverse-coded items on Prosocial Behavior factor model [39]^d,e^ | 645.99 (258)^***^ | .038 (.034-.042) | .89 | .87 | 1.34 |
| 4) Bifactor model for 5-factor 1a with uncorrelated factors [36]^f^ | Model not identified. Problem with the Difficulties factor. | | | | |
| 5) 5-factor, second-order Difficulties factor model [17] | 978.45 (270)^***^ | .052 (.049-.056) | .78 | .76 | 1.70 |
| 6) 6-factor with Prosocial Behavior + reverse-coded items loading on a sixth Positive factor [17] | Model not identified. Problem with the Positive factor. | | | | |
| *df* =Degrees of freedom. CI = Confidence interval. ^***^ *p* < .001. WLSMV estimation. Theta parameterization. Acceptable fit includes RMSEA < .05 or at most < .08 if baseline or null model RMSEA is < .158 [75]. Other CFA fit measures include non-significant (*p* > .05) χ^2^, CFI and TLI > .90 and preferably > .95, and WRMR around 1.0 [73, 74, 76].  ^a^Problem with item 17 “Kind” on the Prosocial Behavior factor. Item 17 “Kind” fixed to one on Prosocial Behavior.  ^b^Correlating item residuals: 1 “Considerate” - 5 “Tantrum”; 1 “Considerate” - 9 “Caring”; 4 “Shares” - “11 “Friend”; 6 “Loner” - 23 “Adults best”; 9 Caring”- 20 “Help out”.  ^c^Best fitting model.  ^d^Correlating item residuals: 1 “Considerate” - 9 “Caring”; 4 “Shares” - 11 “Friend”; 6 “Loner” - 23 “Adults best”; 9 “Caring” - 20 “Help out”.  ^e^Problem with item 9 “Caring” on the Prosocial Behavior factor. Item 9 “Caring” fixed to one on the Prosocial Behavior factor.  ^f^Problem with item 7 “Obeys” on the general Difficulties factor. Item 7 “Obeys” fixed to one on the Difficulties factor. | | | | | |
