# Supplementary material for: Assessing the factor structure of the Spanish language parent Strengths and Difficulties Questionnaire (SDQ) in Honduras
Source: PLoS One. 2019 Mar 28;14(3):e0214394. doi: 10.1371/journal.pone.0214394 (PMC6438563; doi:10.1371/journal.pone.0214394)
Supplement: S2 Table — (DOCX) [file pone.0214394.s003.docx]

| **S2 Table. Confirmatory Factor Analysis of the Best Fitting Previously Identified Model, 1c) 5-Factor + 5 Correlating Residuals^a^ + Cross-Loading Reverse-Coded Items on the Prosocial Behavior Factor [32, 39], with the Parent or Teacher Spanish Language SDQ by Child and Respondent Gender** | | | | | |
| --- | --- | --- | --- | --- | --- |
| **SDQ Factors & Abbreviated Item Names** | **Full Sample**  **(n = 967)** | **Children^b^** | | **Respondents^b^** | |
|  |  | **Boys**  **(n = 490)** | **Girls**  **(n = 477)** | **Male^c^**  **(n = 136)** | **Female**  **(n = 831)** |
|  | **SFL (SE)** | **SFL (SE)** | **SFL (SE)** | **SFL (SE)** | **SFL (SE)** |
| EMOTIONAL SYMPTOMS |  |  |  |  |  |
| 3 “Somatic” | .52 (.04) | .44 (.05) | .60 (.05) | n/a | .53 (.04) |
| 8 “Worries” | .61 (.03) | .63 (.05) | .59 (.05) | n/a | .63 (.03) |
| 13 “Unhappy” | .72 (.03) | .76 (.05) | .70 (.04) | n/a | .74 (.03) |
| 16 “Clingy” | .68 (.03) | .63 (.05) | .74 (.04) | n/a | .68 (.03) |
| 24 “Afraid” | .62 (.03) | .57 (.05) | .66 (.04) | n/a | .61 (.04) |
| CONDUCT PROBLEMS |  |  |  |  |  |
| 5 “Tantrum” | .48 (.04) | .46 (.05) | .50 (.06) | n/a | .45 (.04) |
| 7 “Obeys” (R) | .17 (.08) | .15 (.11) | .13 (.12) | n/a | .20 (.08) |
| 12 “Fights” | .70 (.04) | .65 (.05) | .74 (.05) | n/a | .73 (.04) |
| 18 “Lies” | .59 (.04) | .54 (.05) | .65 (.05) | n/a | .60 (.04) |
| 22 “Steals” | .59 (.07) | .61 (.11) | .60 (.09) | n/a | .56 (.07) |
| HYPERACTIVITY |  |  |  |  |  |
| 2 “Restless” | .51 (.05) | .58 (.07) | .44 (.07) | n/a | .52 (.05) |
| 10 “Fidgety” | .67 (.05) | .69 (.07) | .64 (.07) | n/a | .67 (.05) |
| 15 “Distracted” | .49 (.05) | .47 (.07) | .51 (.07) | n/a | .52 (.05) |
| 21 “Reflect” (R) | .13 (.06) | .15 (.08) | .09 (.09) | n/a | .15 (.06) |
| 25 “Attends” (R) | .28 (.07) | .20 (.10) | .32 (.10) | n/a | .32 (.07) |
| PEER PROBLEMS |  |  |  |  |  |
| 6 “Loner” | .30 (.05) | .29 (.07) | .24 (.07) | n/a | .30 (.05) |
| 11 “Friend” (R) | .12 (.07) | -.05 (.10) | .13 (.09) | n/a | .10 (.07) |
| 14 “Popular” (R) | .08 (.06) | -.09 (.10) | .10 (.08) | n/a | .04 (.07) |
| 19 “Bullied” | 1.00 (.09) | .62 (.10) | .59 (.09) | n/a | .67 (.07) |
| 23 “Adult best” | .20 (.05) | .12 (.06) | .23 (.06) | n/a | .20 (.05) |
| PROSOCIAL BEHAVIOR |  |  |  |  |  |
| 1 “Considerate” | .73 (.06) | .70 (.08) | .71 (.09) | n/a | .71 (.07) |
| 4 “Shares” | .01 (.06) | -.07 (.07) | .06 (.08) | n/a | .02 (.06) |
| 9 “Caring” | .16 (.06) | .10 (.08) | .21 (.09) | n/a | .21 (.07) |
| 17 “Kind” | .31 (.06) | .28 (.08) | .32 (.08) | n/a | .34 (.07) |
| 20 “Help out” | .37 (.6) | .32 (.08) | .37 (.08) | n/a | .38 (.06) |
| 7 “Obeys” (R) | -.49 (.07) | -.52 (.11) | -.50 (.11) | n/a | -.44 (.08) |
| 11 “Friend” (R) | -.43(.07) | -.61 (.09) | -.35 (.10) | n/a | -.42 (.08) |
| 14 “Popular” (R) | -.47 (.06) | -.58 (.10) | -.45 (.09) | n/a | -.51 (.07) |
| 21 “Reflect” (R) | -.44 (.05) | -.36 (.07) | -.51 (.07) | n/a | -.44 (.06) |
| 25 “Attends” (R) | -.50 (.06) | -.45 (.09) | -.58 (.08) | n/a | -.47 (.07) |
| SFL = Standardized Factor Loading. SE = Standard Error. n/a = Not available. R = Reverse coded. WLSMV estimation. Theta parameterization. Problem with item 17 “Kind” on Prosocial Behavior factor. Item 17 “Kind” fixed to one on Prosocial Behavior factor  ^a^Correlating item residuals: 1 “Considerate” - 5 “Tantrum”; 1 “Considerate” - 9 “Caring”; 4 “Shares” - 11 “Friend”; 6 “Loner” - 23 “Adults best”; 9 “Caring” - 20 “Help out”.  ^b^We conducted measurement invariance testing with the best fitting model 1c between male and female caregiver respondents and male and female children. Due to issues with starting values, starting values equal to half the variance for each item were added to first the boys’ then the girls’ models, and to models for male adult respondents. However, the estimated covariance matrix was non-invertible and neither configural or scalar models were able to be estimated for either gender group, indicating model 1c varied based on gender for both children and respondents. These results also suggest that considering RMSEA as the only goodness of fit index, even when baseline RMSEA < 0.158 [74], may not provide a model with adequate fit for measurement invariance testing. Notably, the paper by Gomez and Stavropoulos that argued for relying on baseline RMSEA for assessing CFA model goodness of fit did not test any SDQ model for measurement invariance between different groups [46].  ^c^Item 7 “Obeys” fixed to 1 on the Prosocial Behavior factor. Continued problem with Prosocial Behavior factor. | | | | | |
